# Facing Change and Uncertainty: Lessons Learned from Autistic Children and their Families During the COVID-19 Pandemic

**DOI:** 10.1007/s10803-024-06656-0

**Published:** 2024-12-02

**Authors:** Farah Mgaieth, Melanie Palmer, Tony Charman, Emily Simonoff

**Affiliations:** 1 Forensic and Neurodevelopmental Sciences, King’s College London, Institute of Psychiatry, Psychology & Neuroscience, London, UK; 2 Department of Child and Adolescent Psychiatry, King’s College London, Institute of Psychiatry, Psychology & Neuroscience, London, UK; 3 Service for Complex Autism & Associated Neurodevelopmental Disorders, South London and Maudsley NHS Foundation Trust, London, UK; 4 Department of Psychology, King’s College London, Institute of Psychiatry, Psychology & Neuroscience, London, UK

**Keywords:** Autism, COVID-19, Mental health, Children, Parents

## Abstract

The COVID-19 pandemic presented a great challenge for individuals around the globe, and particularly for vulnerable populations such as autistic children. This qualitative study explored the experience of autistic children (both verbal and minimally verbal) and their families during the pandemic in August-October 2020 through the lens of 18 parents recruited from an opportunistic follow-up of a randomized controlled trial. Findings revealed that the pandemic was detrimental to the mental health of most parents. School closures, disrupted routines and concerns of the virus were believed by parents to be particularly responsible for increased in their child’s behaviour that challenges and anxiety, resulting in changes in acquired skills and development of tics for some. However, other parents reported that increased one-to-one interaction with their child improved their social interaction and communication. Additionally, families felt more able to cope with the situation when supported by their partner, support services and schools. The findings highlight the challenges and benefits experienced by families with an autistic child during the pandemic. They provide valuable insights into potential areas that warrant attention when preparing for future emergencies. Enhancing our ability to respond to the needs of autistic children and their families, and establishing policies that can support their well-being should be prioritised to effectively address future challenges.

## Introduction

As a consequence of the COVID-19 pandemic, the lives of individuals, particularly those with special educational needs and their families, were faced with considerable challenges. The implementation of lockdown restrictions and social distancing guidelines during the COVID-19 pandemic caused substantial disruptions to families’ daily lives. In April 2020, 90% of school-aged children worldwide were out of traditional education (Lee, 2020). The UK Government introduced school closures for the majority of children and young people in March 2020. Schools remained closed for most pupils until June 2020, except for children of key workers and vulnerable children. Even with full physical reopening of schools in September 2020, secure measures such as class-sized ‘bubbles’ remained in place. In addition to school closures, nationwide restrictions during this period included limitations on outdoor activities, with people allowed to leave their homes only for essential purposes such as grocery shopping, exercise, and medical needs. Social gatherings were prohibited, and public spaces were heavily regulated.

Several studies have investigated the impact of these disruptions on children and young people’s mental health yielding mixed evidence. While a few studies have reported that a majority of children experienced improved mental well-being or no change during the first lockdown in the UK (Soneson et al., 2023; Widnall et al., 2020), others have indicated an increase in mental health problems in children (e.g., in depression symptoms) (Bignardi et al., 2020; Mansfield et al., 2021; Thorisdottir et al., 2021), especially in those with special educational needs and neurodevelopmental conditions (Guzman Holst et al., 2023). A large-scale longitudinal study tracking the trajectories of mental health problems of children aged 4 to 16 years over 13 months of the pandemic, suggested that those with pre-existing special educational needs or neurodevelopmental conditions were most at risk of increasing or persistent emotional, conduct, hyperactivity and inattention difficulties compared to other children (Guzman Holst et al., 2023).

Autistic children (we use the terms “autism” and “autistic” to refer to autism spectrum disorder throughout this article as this was reported to be the preferred terminologies by the UK autism community (Kenny et al., 2016)) were particularly vulnerable to the changes brought by the pandemic due to the unpredictable nature of the circumstances (Aishworiya & Kang, 2021). Autism is characterised by social and communication difficulties as well as restrictive/repetitive behaviours and interests (American Psychiatric Association, 2013). Between 33 and 55% of autistic children have an intellectual disability (ID) (Charman et al., 2011; Maenner et al., 2020). Additionally, the manifestation of autism is often further complicated by the presence of emotional and behavioural disorders, chronic medical conditions, and other developmental impairments (Parker & Killian, 2020). Notably, two-thirds of autistic children with special healthcare needs have four or more co-occurring conditions, in contrast to just 13% among those without autism (Karpur et al., 2019). Thus, they present with greater healthcare needs than neurotypical children, may require more intense and consistent interventions from community-based services, and may be less resilient to the effects of COVID-19 (Aishworiya & Kang, 2021). Adapting to new routines due to school closures and frequent changes in public health guidance is likely to disproportionally affect autistic children, especially considering that intolerance to uncertainty has been linked to mental health difficulties (Boulter et al., 2013; Pickard et al., 2020).

In the UK, there is limited evidence about the impact of the COVID-19 on the mental health of autistic children. Autistic children presented with more depression and anxiety symptoms during lockdown compared to those with other special educational needs and disabilities (Toseeb & Asbury, 2023), with lower adaptive functioning measured pre-pandemic shown to be associated with increased behavioural/ADHD symptoms during the pandemic (Palmer, Chandler, et al., 2023). Two systematic reviews showed that behavioural symptoms were the most affected during the pandemic with parents reporting an increase in old behavioural patterns, followed by a worsening of sleep patterns (Dal Pai et al., 2022); Patel et al. (2023), and increased social withdrawal, loneliness, mood fluctuations and anxiety (Patel et al., 2023). While most studies focus on mental health difficulties, the pandemic may have had significant educational setbacks for autistic children who were already presenting with a higher incidence of difficulties with academic skills pre-pandemic compared to their neurotypical peers (Baten et al., 2023) and with their parents reporting feeling less satisfied with the school measures during the pandemic.

These challenges have amplified the demands on parents/carers to support their children’s educational and mental health needs while also navigating shifts in family dynamics. Pre-pandemic, parents of autistic children were already expected to manage additional behaviours that challenges and reported more daily hassles (e.g., continually cleaning up messes) compared to parents without an autistic child (Davis & Carter, 2008; Quintero & McIntyre, 2010). The abrupt shift to remote learning, combined with the need to provide consistent therapeutic support at home due to the closure of schools and in-person services, added to the caregiving load (Lee et al., 2021). Parents found themselves juggling competing responsibilities from both school and home working commitments, with one of their biggest worries being that their child will fall behind in education (Asbury & Toseeb, 2022; Canning & Robinson, 2021). This situation was further complicated by the need to adapt to new family dynamics, such as autistic children spending extended periods with their siblings and/or being separated from other adults involved in their care (Tokatly Latzer et al., 2021).

Moreover, parents of autistic children reported increased intrafamilial burden, arguments, aggression, and physical conflicts during this period (Furar et al., 2022; Isensee et al., 2022). Even prior to the pandemic, these parents experienced higher stress related to parental responsibilities compared to parents of neurotypical children (Craig et al., 2016; Hayes & Watson, 2013), with greater stress associated with mental health problems in children (Yorke et al., 2018). Pandemic-related stressors likely exacerbated existing stress associated with parental responsibilities. Parents of autistic children reported increased levels of stress and anxiety due to financial difficulties as many had changes in their employment and their children at home all day long during the pandemic (Yilmaz et al., 2021). Higher pre-existing parental distress levels among these parents were associated with more pronounced parental mental health symptoms during the pandemic (Palmer, Chandler, et al., 2023), with overall greater psychological distress compared to the general population, especially for feelings of panic when thinking about COVID-19 (Kalb et al., 2021). On the other hand, Toseeb and Asbury (2023) found that psychological distress and well-being levels of these parents remained stable at the beginning of the first lockdown and after face-to-face teaching resumed for all children.

A comprehensive study suggests that the probability of experiencing extreme pandemics similar to COVID-19 within one's lifetime currently stands at approximately 38% (Marani et al., 2021). Additionally, rapid changes in climate as well as increasing frequency and severity of environmental disasters such as floods or earthquakes may influence the well-being of children and their families, particularly those with disabilities (Mann et al., 2021). Thus, it is essential that we learn from previous experiences to be prepared for and respond to future challenges associated with potential pandemics and other major disasters.

While numerous studies have quantitatively assessed the impact of COVID-19 on the mental health of autistic children and their parents (Alonso-Esteban et al., 2021; Milea-Milea et al., 2023; Toseeb & Asbury, 2023), the current study adopts a qualitative approach to explore lived experiences of families in the UK recruited from an existing cohort during the pandemic. This qualitative approach intends to gain insight into the specific challenges as well as positive factors influencing the life of autistic children and their families as a result of the pandemic. This detailed information will be particularly valuable to understand how to support best families with autistic children in the event of another pandemic or other disasters. Thus, the aim of the study was to extend existing knowledge by giving a voice to parents of autistic children and explore their families’ experience of the pandemic from their perspective. This will be captured by primarily addressing children’s mental health, access to education and other support services, managing/coping mechanisms in the context of the pandemic, in addition to parents’ well-being.

## Methods

### Participants

A sub-sample of parents with autistic children who had taken part in the Autism Spectrum Treatment and Resilience (ASTAR) study (ISRCTN91411078) (Charman et al., 2021; Palmer et al., 2019), as part of the Improving Autism Mental Health project, were invited to complete online semi-structured interviews during the COVID-19 pandemic (of those invited, *n* = 18 completed the interviews, mean age of child = 8.74 years, SD = 1.68). The ASTAR study was a non-randomized feasibility study followed by a pilot randomized controlled (RCT) of two novel parent-mediated group interventions conducted between 2017 and 2019. The target intervention (Predictive Parenting) aimed to reduce common co-occurring difficulties, such as emotional and behaviour problems, in autistic children. This was compared to an attention control condition which focused on psychoeducation about autism. See Palmer, Chandler, et al. (2023) for further details about the inclusion criteria.

### Procedure

**Fig. 1:**
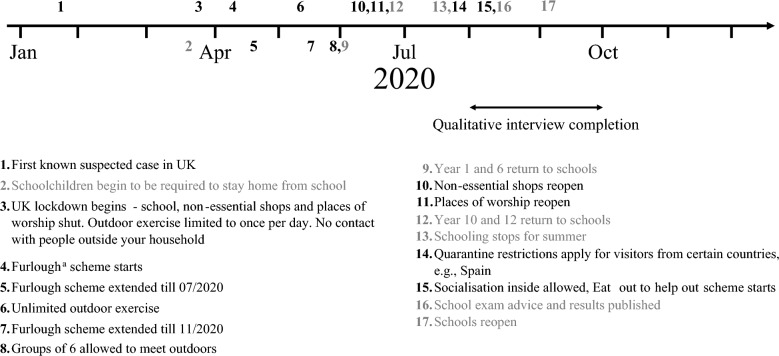
Key UK COVID-19 related regulations between January and October 2020

ASTAR cohort participants were re-contacted and asked to complete an online questionnaire from August 2020 asking about their experience of the pandemic (for further information, see Palmer, Chandler, et al. (2023)). A sub-set were then invited to participate in a follow-up interview. Purposeful sampling was used to ensure we sought the views of participants who reported a range of pandemic-related experiences on the online questionnaire, namely, positive, mixed and negative experiences. Additionally, participants were sampled across two other variables: whether the child was minimally verbal or verbal, and whether they had a garden during lockdown as we expected these two factors to be important contributors to pandemic-related experiences. The ADOS-2 module was used for verbal ability stratification (minimally verbal = module 1, defined as pre-verbal or using single words; verbal = module 2 or 3, defined as using phrases or fluent speech) (Lord et al., 2000). A total of 25 participants who completed the questionnaires and gave consent for interview were invited to complete online semi-structured interviews between August and October 2020 (see Fig. 1 for a timeline of COVID-19 related regulations). Of those, 18 parents/carers (17 mothers and 1 father) of autistic children (9 minimally verbal and 9 verbal) completed an interview. Sample characteristics are provided in Table 1. All interviews were conducted by either FM or MP (who were trained to ensure consistent interview style prior to conducting the interviews and who did not have a previous relationship with the interviewees) over video-conferencing software at a time convenient for the family and were recorded and transcribed verbatim assisted by the Microsoft Teams auto-capture function. Parents were given the opportunity to review and correct transcripts prior to analysis. Ethical approval for the original pilot RCT was granted from NHS Camden and Kings Cross Research Ethics Committee (16/LO/1769). The current study was reviewed and given ethical approval from the Psychiatry, Nursing and Midwifery Research Ethics Subcommittee at King's College London (REMAS ref: 19,146, ethical clearance ref: HR-19/20–19146). All parents gave written informed consent.

**Table 1:** Sample Characteristics by Verbal Ability

	Verbal (*n* = 9)	Minimally verbal *(n* = 9)
Baseline characteristics		
Age: mean in years, (SD), range	9.6 (1.0), 8.0–10.9	7.7 (1.6), 6.2–11.4
Sex: n (%)	6 Male (66.7)	9 Male (100)
Ethnicity: n (%)		
White	5 (55.5)	5 (55.5)
Black African/Caribbean	2 (22.2)	1 (11.1)
Asian/Asian British	0 (0.0)	1 (11.1)
Mixed	2 (22.2)	2 (22.2)
School type: n (%)		
Mainstream + Unit in Mainstream	8 (88.9)	3 (33.3)
Specialist	1 (11.1)	6 (66.7)
Parental education^a^ – highest level: n (%)		
No formal qualifications	0 (0.0)	1 (11.1)
NVQ, City and Guilds, or equivalent	1 (11.1)	1 (11.1)
A levels, or equivalent	2 (22.2)	2 (22.2)
Undergraduate degree	3 (33.3)	1 (11.1)
Postgraduate degree	3 (33.3)	4 (44.4)
Parental^a^ employment during the pandemic (previous 2 weeks): n (%)		
Working usual hours (full-time or part-time) from home	4 (44.4)	3 (33.3)
Working more hours than usual	1 (11.1)	0 (0.0)
Working fewer hours than usual	1 (11.1)	1 (11.1)
Was not working outside the home	1 (11.1)	5 (55.5)
Furloughed due to COVID-19	2 (22.2)	0 (0.0)
Level of financial concern during the pandemic (previous 2 weeks): n (%)		
Finding it quite/very difficult	1 (11.1)	1 (11.1)
Just getting by	4 (44.4)	4 (44.4)
Doing alright	3 (33.3)	1 (11.1)
Living comfortably	1 (11.1)	3 (33.3)
Child access to education during the pandemic (previous 2 weeks): n (%)		
No face-to-face education	5 (55.5)	7 (77.8)
Some face-to-face education	4 (44.4)	2 (22.2)
Home environment: n (%)		
Comfort		
Very problematic	1 (11.1)	1 (11.1)
Somewhat problematic	2 (22.2)	1 (11.1)
Fairly comfortable	1 (11.1)	3 (33.3)
Very comfortable	5 (55.5)	4 (44.4)
With access to a personal garden/outside space	8 (88.9)	9 (100)
ASTAR study treatment allocation: n (%)		
Predictive Parenting	4 (44.4)	6 (66.7)
Psychoeducation	5 (55.5)	3 (33.3)

^a^Relates to responding parent/carer

### Measures

Semi-structured interviews lasted between 32 and 71 min (average duration was 50 min) and covered the following areas:

1. The family’s experience of the COVID-19 pandemic and impacts on the child and family (follow-up prompts included the impact on the child’s emotions and behaviours, the child’s education, family life, any positive impacts, and changes over the course of the lockdown)
2. Factors that had helped the family cope with the impact of the pandemic (follow-up prompts included asking about the family’s personal circumstances, formal and informal support networks, and professional help).

The interview guide was collaboratively developed with the principal investigator and other team researchers and main themes and questions were monitored and adjusted throughout the interviewing process, and are provided in the supplementary materials. Qualitative views on the usefulness of the intervention received as part of the original ASTAR pilot RCT were also enquired about but are not discussed here as they are reported in Palmer, Carter Leno, et al. (2023).

### Data Analysis

Data collected from the semi-structured interviews were analysed using an inductive thematic analysis approach based on grounded theory methods (Palinkas, 2014) in NVivo. The process involved multiple readings of the transcripts, from which a coding scheme was developed which was also informed by the emerging literature detailing the impacts of the COVID-19 pandemic on autistic children. The coding scheme identified themes and was first applied to the raw data by FM. Double coding of the data was subsequently done on 4 interviews by MP to ensure accuracy and consistent application of the scheme. Following this, both researchers jointly reviewed the data and codes to confirm that the identified themes comprehensively represented the dataset and were accurately linked to each coded extract. Any irrelevant codes—those that did not directly relate to the research questions or key emerging themes—were excluded through an iterative process of refinement. In cases of disagreement, discussions with the principal investigator (ES) were conducted until consensus was reached. ES also oversaw the coding process, ensuring that the analysis was methodical and rigorously applied. Themes that emerged are reported in the Results section, with each parent assigned an anonymous code to ensure confidentiality.

## Results

### Impact on Children

#### Behaviour that Challenges

Just over half of the parents (*n* = 10) reported that their child displayed an increase in behaviour that challenges, such as being irritable or moody, temper tantrums, being physically aggressive, or throwing/damaging objects. Increases in behaviour that challenges were believed by parents to be related to: (1) the child’s lack of understanding of the situation and not being able to do what they want, (2) not receiving as much stimulation at home in comparison to what would usually be provided at school, (3) increased lockdown-related screen time, and (4) boredom. This resulted in a lot of stress for parents, and in some cases increased conflict between children and their siblings, as well as substantial costs to repair and replace damaged property (e.g., “*We had to also pay to pave the side of our garden. He would take those stones and throw them. We ended up paying somebody which cost hundreds.*”(P9)). Some parents reported that increases in behaviour that challenges were seen initially when lockdown was first imposed but decreased over time as their children got more used to the situation. Other parents reported the opposite pattern, with children becoming increasingly frustrated and angry as time went on:

“His behaviour went downhill. He was very angry, especially midway through to the end. He became very aggressive. He was throwing things a lot. He destroyed a lot of furniture and his iPad about two or three times. … His room has dents on the wall from where he's taken his toys, and he's thrown it against the wall. … I mean he was throwing things from the second he woke up all the way through the day and it was really, really challenging.” (P9)

Additionally, parents of three minimally verbal autistic children reported that their child eloped on at least one occasion during lockdown, believed to be due to boredom or trying to take themselves to school. For two parents, their child had eloped pre-pandemic and this occurred even though security measures like window locks and high fencing were in place. In all cases, eloping resulted in involvement with statutory services from police and/or social care (of which the experience of social care support varied from negative to positive depending on whether it was provided by disability social workers or not), additional private interventions (more locks, security fencing) and stress for parents.

“During the pandemic [early lockdown], he escaped and the police had to bring him back. … He managed to sneak out the back gate. … The police got called so quick because he had run out naked. … I think he was trying to take himself to school. … About three weeks ago, that's when he escaped again and he almost got hit by a car. … I've had to borrow a lot of money off my Nan and we’re going to get the front garden all security fenced.” (P11)

#### Anxiety

Ten parents of both verbal and minimally verbal autistic children reported an impact of the pandemic on their child's anxiety. Most parents reported an increase in anxiety, related to the concerns about catching the virus or due to changes in structure, routine and the unpredictability of the situation. This led to some children refusing to leave the home, becoming disobedient, controlling and aggressive, and washing excessively. Several parents also reported an intensification in a restricted interest or fixation on a certain activity.

“She likes to be in control of her situation, her life, but she does benefit from structure, providing it isn't too obvious. The lack of going to school and then the restrictions around not being able to see friends and family, the unknown I guess of when this is going to go back to normal. I think not seeing family was a huge issue, and her anxiety was extremely high throughout the whole of lockdown really.” (P4)

On the other hand, parents of three children reported a decrease in their child’s anxiety. All of these parents reported that less anxiety was related to not having to attend school and the social demands of that environment, sometimes leading to less behaviour that challenges. For example, one parent stated:

“Term time is usually when he sort of displays more angry outbursts or meltdowns and that tends to be when he comes home from school. During lockdown, you didn't see as much of this because he wasn't anxious. No self-harm during that period - that's always been a school term thing.” (P7)

#### Decrease in Developmental Skills

Several parents of both verbal and minimally verbal children reported lockdown-related developmental changes in their child's behaviour (*n* = 2) such as “acting a lot younger”, as well as toileting issues (*n* = 2), or returning to sleep with parents (*n* = 1). One parent also observed a decline in their son’s interaction skills, impacting his friendships, while another parent expressed that their child “*regressed in every way*” (P9) with their speech becoming incomprehensible.

“When we spent the whole day and got him to write three lines and he was hiding under the table… I hadn’t seen that since he was in the early stages of primary school. That’s what he used to do. He would be like “I’m not doing anything”, he would cover his head and stuff.” (P2)

#### Social Distancing

Four parents reported their children had difficulties with social distancing due to poor impulse control despite two of them fearing the virus; a situation difficult for parents who had to stay with their child at all times and go to quiet places to ensure they “*would not do anything stupid*” (P1):

“[My daughter] is really bad at social distancing,[she] does not understand what social distancing is. And her impulse control is very poor, so she touches everything and she is in people’s faces and spaces all the time.” (P6)

#### Communication and Interaction

One parent noted that their child developed inappropriate language as a result of difficulties coping with the new school dynamic (e.g., with the introduction of class-sized ‘bubbles’) e.g., with on one occasion, the child threatening to harm her classmates. Two other parents also reported increased frequency of vocal and motor tics which impacted their child’s communication:

“She’s always had tics and they got more and more pronounced to the point where her CAMHS (Child and Adolescent Mental Health Services) consultant has now said it looks like … it’s consistent with Tourette syndrome […] because she is tic-ing so much that she can’t actually express herself. The vocal tics are so pronounced that she can’t actually speak.” (P6)

However, three parents noticed improvement in their child’s communication and interaction perceived to be due to increased one-to-one adult interaction, with two of them reporting an increase in eye contact:

“All of a sudden, like he's never done this before, but he started throwing the water at me and then we started having a water fight, and he started running. He was so much more interactive than he's ever been.” (P11)

### Impact on Parents

#### Mental Health

Almost all parents reported a negative impact of the pandemic on their mental health, reporting increased stress and worry, feelings of exhaustion and reduced sleep. Some parents reported somatic health impacts associated with stress, such as headaches, eczema outbreaks, and weight gain.

“I was at home with the boys the whole time everyday all day and night and it was exhausting. Some days - my eyes - I just couldn't keep open. [They were] so heavy and it was a struggle to stay up the whole day. It was just quite isolating as well, not being able to do anything.” (P9)

#### Personal Time and Work Commitments

Half of the parents who were interviewed expressed that the pandemic had impacted their personal time. Most reported that it had increased time pressure, with domestic duties and having time for themselves being negatively impacted. Several parents reported that support from a partner or activities enabled them to have some alone time.

All but two parents reported that the pandemic had impacted on their own or their partner's work/studies. Where parents continued to work, most reported challenges with managing the demands of work and home-schooling leading to feelings of guilt due to feeling unable to fulfil either role effectively. As a result, some parents re-scheduled their working hours to meet demands and some reduced their hours or were unable to complete aspects of their studies both of which had a negative financial impact. Other parents reported benefits of their ability or their partner's ability to work from home, with less commuting making them more available to their children.

“We were very fortunate. [My husband] wouldn't usually work from home, but he does now and will do like everyone for the foreseeable future. So he worked the whole way through and actually that part has worked really well for us because the children are much happier knowing that he's upstairs and although he is working and there are times they can't talk to him, I think it does reassure them that he isn't far away.” (P4)

#### On Relationships

Some parents reported the stress associated with the pandemic and lockdown put strains on family relationships, with increased conflict between siblings and co-parents, *“I think from a mental health perspective, my mental health and my husband’s and our relationship has never been worse. […] We are still holding it together but I think the cost is enormous”* (P6). Others reported positive changes in family relationships, such as increased support from a co-parent and improved knowledge of how to support and interact with the child, due to spending more time together as a family.

### Impact on Siblings

Half of the parents discussed the impact of the lockdown on siblings and their relationship with the autistic child. Six parents noted that siblings frequently witnessed increased aggressive or disruptive behaviours, which negatively affected their emotional well-being. Additionally, some siblings experienced heightened aggression from their autistic sibling, resulting in a deterioration of their relationships:

“They knew when he was going to hit them. It wasn’t really a hard beating but they were scared when they see him coming for them and say, ‘Mummy, come to rescue me’.” (P13).

On the other hand, two parents reported no significant changes in sibling dynamics, and one parent even observed an improvement, citing that the extended time together during lockdown had fostered moments of positive interaction.

## Support from School and Other Services

Some children were able to have physical access to their usual school setting a few hours or days a week due to having special needs or parents being key workers. Parents reported that children going to school during the lockdown were mostly provided with childcare rather than educational support. One mother believed that school consciously avoided providing educational support in order to prevent having too many children attending:

“The verbal and non-verbal messages the school kept sending was very much resenting having to come in to look after children as key workers. […] [My daughter] was going to school, but then the school was like ‘you know when she comes here, she just sits in place. We are just providing almost baby care, childcare’. They felt if they provided any additional support then more people would bring their kids or I don't know what they thought.” (P3)

Parents who reported having a more positive experience with the school felt supported and could communicate regularly with the teacher and/or other school staff members (e.g., occupational therapists, special educational needs coordinator). On the other hand, some parents felt that the school could have been more supportive with their child’s homework with some parents only receiving homework without much contact from the school staff:

“All the support she had in school disappeared. […] Because everything had stopped, in its place I was being sent lots of PDFs. […] I have a stack of PDFs that’s like that and two children. In school, [my daughter] got one to one support and suddenly I’m one to two. So what am I supposed to do? […] Overnight, I was supposed to be a teacher, occupational therapist, speech and language therapist, physical therapist and a mental health specialist monitoring her destructive behaviour.” (P6)

Furthermore, due to the nature of the restrictions, teachers were unable to prepare children to transition from one school year to another despite the need for greater preparation for this population (Makin et al., 2017). When back to school, children were thus thrown into a new environment which parents believed exacerbated the level of anxiety of their children and was described as “traumatic” by one mother.

Most parents reported encountering difficulties with different support services. All the specialist support in education, mental health, social services and council (local authorities) prior to the lockdown suddenly stopped in March 2020. During summer, some parents were constrained to pay for private tutors to provide educational support to their children to mitigate the impact of the pandemic learning losses, or to do local activities to provide some respite for themselves and their children.

During the early phases of the pandemic, panic buying of foods and limited access to online delivery grocery slots became a concern for several families with a child with a restricted diet. While five parents reported that their child became more adventurous with food and was eating a wider variety of food due to the unavailability of their favourite foods, some parents encountered significant difficulty to replace them. As one parent said:

“[My son] has got such a limited diet. He’s very reliant on things like McDonald's and then obviously as soon as that closed then he was just back on his rice cakes and maybe some toast. So basically his warm meal of the day was taken from him. We tried our own, we tried doing our own chips- sometimes he ate them, sometimes he didn’t eat them. We tried our own chicken nuggets. That wasn't very successful because he's very sensory, he can look at a nugget and if it doesn’t look quite right then he won't eat it.” (P15)

## Educational Attainment and e-Learning

Remote learning had been a positive experience for some children especially for those able to receive one to one support either from their parents or personal tutors. Children benefitted from a structured environment established by parents to help them transition from one activity to another, as well as from receiving a more flexible educational approach allowing them to have several breaks during the day with learning methods adapted to the child’s needs. Parents reported that working closely with their child allowed them to get a better understanding of their child’s weaknesses and strengths and what they need to work on to improve their educational experience.

"He knew that Monday to Friday there's learning at home with mum which starts at 9:00 o'clock in the morning and he had a plan on the wall. Exactly what subjects he's going to be doing each day, when is his break, when his lunch is and when he comes back after lunch, what else he has to do and when it's finished." (P14)

However, remote learning was challenging for many families especially for working parents who had to balance their work demands and their child’s home schooling. Parents with more than one child at home felt that they had to “*split themselves*” in order to assist their children equally with the guilt of not being able to provide a full-time support to one child only especially for children who struggled to work independently. Difficulties also arose especially at the beginning of the lockdown when children were suddenly unable to attend school. Implementing a new learning experience for children who often display rigid thinking, without transition, was a challenging experience for several families. Some children with difficulty understanding the reason behind this sudden change and for not being able to go to school anymore were left wondering why their parents suddenly became their teachers. Blending home and school together was reported to be difficult by some parents with children refusing to listen to them:

“When it came to actually home learning and home education, I mean… just absolute refusal. It was a sort of demand avoidance. You know ‘who are you?’, ‘you’re not my teacher’, ‘why are you making me do this?’” (P6)

## Discussion

The current study explored the consequences of the COVID-19 pandemic on autistic children and their families in the UK. The study highlights both the challenges and benefits of the first months of the lockdown using an in-depth qualitative approach, providing a unique perspective on the adaptive behaviours and resilience shown by families. These findings contribute to understanding how a population disproportionally impacted by emergencies can experience mixed outcomes, offering valuable insight for shaping preventive strategies and improve preparedness when facing future disaster scenarios.

### Mental and Behavioural Health of Autistic Children

During the pandemic, many autistic children were unable to access the services outlined in their Education, Health and Care Plans (EHCPs). Although entitled to full-time school attendance under EHCP, most did not return until June 2020. This aligns with findings from the Children’s Commissioner for England, which reported only 6% of children with an EHCP attended school during lockdown (Children's Commissioner, 2020). For autistic children, already more prone to loneliness and anxiety than their neurotypical peers (Hymas et al., 2022), school closures likely had an especially detrimental effect on their well-being.

In our study, parents identified that their child exhibited heightened anxiety and/or behaviour that challenges during the initial months of the pandemic. This is consistent with other research showing that autistic children experienced greater behavioural changes and anxiety compared to their neurotypical peers and children with other neurodevelopmental conditions (Amorim et al., 2020; Berard et al., 2021; Guller et al., 2022; Hosokawa et al., 2021). Our study also highlights additional behaviours that seemed specific to autistic populations such as reductions in previously acquired developmentally appropriate skills, tic development and in some cases eloping (only in those who are minimally verbal). These changes were believed to be mainly driven by a lack of stimulation, outdoor activities, adequate understanding of the situation, as well as of structure. Considering that intolerance to uncertainty is associated with anxiety in autistic children (Lidstone et al., 2014), routine disruptions may have intensified anxiety and disruptive behaviours. Additionally, some parents reported that their child’s heightened anxiety stemmed from virus-related fears, consistent with findings that autistic youth experience greater concern about COVID-19 and more emotional arousal under stress than neurotypical peers (Corbett et al., 2021).

In contrast, some children presented with reduced anxiety along with improved communication and overall well-being, which parents attributed to increased one-to-one time spent with them. However, for these children, there were no reported improvements in their behaviour. While schools promote behaviour regulation, they can be a challenging setting for some autistic children due to co-occurring mental health conditions (Lai et al., 2019) and susceptibility to bullying (Forrest et al., 2020), potentially explaining the reduced anxiety for some children in the study. Furthermore, COVID-19 disruptions to food access (Aday & Aday, 2020) impacted autistic children with atypical eating behaviours when preferred items were unavailable (Bellomo et al., 2020). Some parents noted this led to a more varied diet, a positive shift also observed in other studies, where a calm home environment encouraged trying new foods (Dhillon-Burrows et al., 2023; Tokatly Latzer et al., 2021).

### Mental Health of Parents

The majority of parents reported higher stress levels and less personal time for self-care, with balancing work and home-schooling, especially in households with multiple children, posing a significant challenge. School closures and the suspension of support services left parents juggling multiple roles to ensure their child's education continued. Some parents found partial relief through partner support, engaging in various activities, and schools that facilitated communication. Our qualitative study also captured the financial burden some parents experienced specifically in relation to their child’s behaviour changes, including the cost of damaged items and home adjustments to ensure safety.

### Implications for Practice and Future Directions

The COVID-19 pandemic disrupted many of the essential services for autistic children and their families, creating significant challenges across educational, behavioural, and healthcare domains. Many of these children, reliant on structured school environments and one-on-one support, were suddenly required to learn at home, imposing additional educational demands on parents despite government policies aimed at maintaining school access for children with special needs. In future emergencies, ensuring that these children continue to receive vital behavioural health and support services will be crucial to help them process changes, adapt to new environments, and reduce potential mental health impacts.

#### Implications for Children

Government and educational institutions should develop robust emergency response strategies to ensure continuous access to essential services for autistic children during crises. A framework that upholds individualized education plans (IEPs), even during school closures, would be essential. Lessons from the pandemic suggest that small class sizes and personalized attention benefited some children; those in smaller groups experienced improved teacher interaction and peer relationships (Smith, 2021). Future emergency preparedness should incorporate these settings to support the educational and emotional well-being of autistic children.

Proactive and consistent family communication is also critical, especially during school closures. Strategies like video tours of school campuses or pre-term visits can help ease transitions back to routine, reducing anxiety and bolstering confidence (Smith, 2021). The pandemic highlighted gaps in healthcare services for children with EHCPs, particularly for those needing multi-professional input. Many behavioural and therapeutic services moved online or were suspended, creating access challenges. To address this, governments should establish integrated service delivery models that combine educational and healthcare support, minimizing reliance on in-person care.

Telehealth (i.e., the use of video technology to access health care services) saw increased use during the pandemic, but its effectiveness for autistic children has been mixed (Bundy et al., 2023). Telehealth remains relatively new for some families, and autistic children may need adaptations like parental involvement, visual aids, and activities that reflect their interests (Ellison et al., 2021; Moree & Davis, 2010). Future investment in telehealth research tailored to autistic populations will be crucial for maintaining service quality in crises. Given that virus fears can be heightened among autistic individuals, accessible resources in various formats could help alleviate these anxieties.

Additionally, the pandemic emphasized the importance of physical activity, as inactivity can negatively impact the quality of life, health, and well-being of autistic children. Physical activities such as walking, stretching, and household chores can promote family involvement, improve motor skills, and help manage behaviour that challenges (Yarimkaya & Esenturk, 2022). Engaging autistic children in regular physical activity offers valuable benefits for their mental and physical health.

#### Implications for Parents and Caregivers

While autistic children are a primary focus, interventions must also address the mental and emotional well-being of caregivers. Studies show that stress levels among caregivers of autistic children increased during the pandemic due to their caregiving responsibilities, lack of respite, and disruptions to normal routines (Manning et al., 2021). A family-centred approach that includes support for parents' mental health and wellbeing, such as access to online counselling, support networks, or respite services, could be beneficial (Bozkus-Genc & Sani-Bozkurt, 2022; Kalb et al., 2021). For example, organising virtual peer support groups or providing subsidies for in-home assistance can help alleviate the burden on caregivers. Fostering strong, community-based networks to provide social and emotional support during emergencies can be vital. Parents reported that connecting with other families facing similar challenges helped alleviate some of their burdens during the pandemic (Bozkus-Genc & Sani-Bozkurt, 2022; Filler et al., 2022; Suresh et al., 2021). Furthermore, training parents on managing their child’s educational and behavioural needs through accessible online resources could be a vital step toward ensuring continuity of care.

## Limitations

The results of the current study should be interpreted in light of several limitations. Findings are from an opportunistic follow-up study involving parents previously enrolled in a RCT of group-based parent-mediated interventions. Although parents of both verbal and minimally verbal children were included for a more representative sample, the eligibility criteria did not require a pre-existing level of problems; thus, some children may have exhibited relatively low levels of disruptive behaviour and anxiety before the pandemic. It is also important to highlight that, while we aimed to capture a representative sample by including parents from various ethnic and educational backgrounds, the sample remains predominantly White. This demographic limitation restricts our ability to draw broader conclusions that fully encompass the experiences of racially and culturally diverse families. Additionally, although we included families living in homes with and without gardens, most participants resided in urban areas within London. This limits the generalisability of our findings, as families living in rural areas may have experienced the pandemic differently. Moreover, the sample predominantly consisted of mothers, with only one father participating, emphasising the maternal perspective on the pandemic experience. Research indicates that female caregivers of autistic children experienced higher perceived stress during the pandemic compared to their male counterparts (Friesen et al., 2021). Additionally, mothers were found to be ten times more likely than fathers to take on homeschooling responsibilities (O'Sullivan et al., 2022). The lack of male perspectives in the current study likely leads to an incomplete understanding of how the pandemic impacted the overall dynamics within families with autistic children.

## Conclusion

The COVID-19 pandemic has exposed the vulnerabilities within both educational systems and healthcare services, while highlighting the importance of preparedness in effectively addressing challenging scenarios, especially when considering autistic children. Findings from this qualitative study indicate the importance of structure and routine for autistic children’s mental health and well-being, and of continuity of education and care. The disruption of these essential elements during the pandemic has highlighted that the provision of individualised support is paramount for minimising adverse mental health outcomes. Furthermore, supporting parents of autistic children may help to mitigate further mental health difficulties which may develop in the face of future emergencies. Implementing strategies that foster resilience and provide resources for both children and their families can enhance coping mechanisms during crises. Policymakers and educational institutions should prioritize the development of integrated service delivery models that combine educational and healthcare support to ensure autistic children continue to receive necessary services, even in challenging circumstances.
